# Statocyst Ultrastructure in the Norwegian Lobster (*Nephrops norvegicus*)

**DOI:** 10.3390/biology13050325

**Published:** 2024-05-07

**Authors:** Marta Solé

**Affiliations:** Laboratory of Applied Bioacoustics, Universitat Politècnica de Catalunya, BarcelonaTech. Rambla Exposició s/n, 08800 Vilanova i la Geltrú, Barcelona, Spain; marta.sole@upc.edu

**Keywords:** statocyst, hair cell, crustaceans, *Nephrops norvegicus*, scanning electron microscopy, transmission electron microscopy

## Abstract

**Simple Summary:**

In this study, we describe the structure of the Norwegian lobster (*Nephops norvegicus*) statocyst using scanning and transmission electron microscopy. These results contribute to our understanding of sound perception systems in an additional crustacean species in order to favor actions for marine noise pollution mitigation to protect the marine fauna.

**Abstract:**

Statocyst anatomy and fine morphology in Norwegian lobster (*Nephrops norvegicus*) are studied for the first time using scanning and transmission electron microscopy. *N. norvegicus* exhibits sensory setae projecting from the statocyst inner cavity floor into a mass of sand granules (statoconia) embedded in a gelatinous substance. The setae are distributed in four areas: a curved field made up of an inner single row and an outer double row that run on a circle around the medial and lateral rim of the central depression, a small setal field in the posterior part, a large setal field, opposite to the small field, and a short row, running internally and lying parallel to the inner single row, next to the small setal field. A study of the fine morphology of the statocyst sensory setae shows that the structure of the setae in the different areas is similar, with a bulb (the proximal portion of the sensillum), a setal shaft, a tooth (the smooth portion of the bulb), a fulcrum (a transverse fold), and filamentous hairs. The hair cells are firmly implanted within the cuticular layer. Although the type of innervation of the statocyst was not determined in the present study, the close taxonomic position of the lobster to that of the crayfish and crab would suggest that the setae in *N. norvegicus* are pure mechanoreceptors rather than sensory cells.

## 1. Introduction

Marine invertebrates, in general, and crustaceans, in particular, are known to use acoustic signals for intra- and interspecific communication. Although sound production was evidenced in two crustacean groups, barnacles (*Cirripeda*) and decapods (*Eumalacostraca*), sound detection is widespread among crustaceans [1,2,3].

Three systems make crustaceans able to detect mechanical disturbance in water/sediment associated with sound waves: a pair of statocysts, chordotonal organs linked to the joints of antennae or legs, and internal and external sensilla [2,4]. To be able to orientate itself in the 3D marine environment, marine invertebrates need a gravity receptor system. These receptors, the statocysts, are common in different groups of marine invertebrates (cephalopods [5], crustaceans [6,7,8], cnidarians [9], and gastropods [10]). Statocysts are fundamental for the regulation of vital invertebrate behavior, including locomotion, posture, balance, and movement in the water column [2,11,12]. In addition, invertebrate statocysts detect sound particle motion rather than the sound pressure and are involved in underwater hearing [12,13]. Lovell et al. found that a sound-evoked response was generated in the statocyst after both ablation of the organ and section of the innervating nerve [7].

In crustaceans, the statocyst consists of a sac-like epidermal invagination of the cuticle located in the basal segment of each antennule in decapods and in the uropod or telson of the tail in mysids and isopods. The basic statocyst structure has similar features in all crustacean species [8]. The crustacean statocyst shows an inner sensory epithelium arranged in two to four rows of hair cells (setae) polarized towards the center of the organ and an overlying statolith, made of agglomerated sand granules [13], which stimulates the sensory setae [13,14]. When the animal changes its position, the statolith deflects the setae, and as a consequence, the afferent neurons innervating the statocyst are stimulated, promoting different responses [11,15] through compensatory movements of the appendages and the body [16].

The structure and morphology of the statocyst sensory epithelium vary depending on the crustacean taxonomical group [11,17]. The statocyst of the crayfish *Orconectes limosus* shows four separate fields of setae: a lateral group in two semicircles, a fusiform medial group, and a single row of proximal setae [18]. The statocyst of the Australian crayfish (*Cherax destructor*) is formed by three fields of setae: a curved field of two setal rows forming a semicircle around the medial and the posterior rim of the central depression, a large triangular lateral field of setae, and a smaller triangular setal field on the medial side of the depression [8]. The blue crab (*Callinectes sapidus*) statocyst exhibits three groups of mechanosensory setae (thread, free hook, and statolith) located in the statocyst canals [19]. Smaller species show smaller statocysts with different setal distribution. Lovell et al. described the fine structure of the prawn *Palaemon serratus*, which exhibits a regular row of setae dividing further into two irregular rows of setae, slightly curved and oriented towards the center of the crescent [7]. The hermit crab *Dardanus calidus* statocyst exhibits four groups of setae distributed along the statocyst inner walls (a curved field made up of two setal rows forming a semicircle around the central depression, a lateral semicircle of setae, a smaller medial semi-circular field, and a single row of setae in the center of the cavity) [20]. The setae in the telson statocyst were studied in some crustacean species [21,22].

Providing precise information on auditory systems in diverse marine species is of importance because of the rising concerns regarding the acoustic impact of anthropogenic noise on marine animals [2]. This concerns have promoted research regarding (i) crustaceans’ capacity to perceive strong vibrations transmitted through a solid [23], including loud anthropogenic noise generated in the marine environment [2,6]; (ii) the structure and physiology of crustacean sensory sound perception systems.

Although both structure and function of some crustacean species’ statocysts are well understood, in the Norwegian lobster (*N. norvegicus*), a species of important commercial interest, they have not been previously described. *N. norvegicus* is widely distributed on soft sediment, commonly at depths of 200–800 m, throughout the North-East Atlantic, from Iceland in the north to Morocco in the south, including the Mediterranean and Adriatic waters. Here, we examine and describe for the first time the micromorphology and ultrastructure of *N. norvegicus* statocyst sensory epithelium using scanning (SEM) and transmission (TEM) electron microscopy. This study aims to better understand the sound perception system in an additional crustacean species in order to favor actions for marine noise pollution mitigation to protect the marine fauna.

## 2. Materials and Methods

### 2.1. Animals

Thirty adult Norwegian lobsters (*N. norvegicus*) of mixed sex, ranging in total length from 13 cm (77 g) to 18 cm (120 g), from South West Scotland, were used for this study (January–March 2023). The animals were kept for five days, prior to the start of the analysis process, in the LAB’s (Laboratory of Applied Bioacoustics, 41°12′57.1″N 1°43′59.0″E) maintenance system, a closed circuit of recirculating water (at 15 °C, salinity 35, and natural oxygen pressure) consisting of 2 mechanically filtered fiberglass-reinforced plastic tanks of 2000 L connected to each other. These maintenance facilities included a physicochemical self-filtration system with activated carbon and sand, driven by a circulation pump.

### 2.2. Scanning Electron Microscopy (SEM) Ultrastructural Analysis

For the SEM analysis, we used the 40 statocysts from 20 animals of both sexes. The lobsters were anaesthetized and sacrificed with an overdose of 2-phenoxyethanol. The statocysts were dissected and placed in a solution of 2.5% S-carboxymethyl–l-cysteine in sodium chloride to hydrolyze the mucus surrounding the statocyst hair sensilla in order to eliminate the statoconia (sand grains). After 2 min, the solution was removed, and the samples were fixed and processed by routine procedures for analysis by SEM. Fixation was performed in glutaraldehyde 2.5% for 24–48 h at 4 °C. The samples were dehydrated in graded alcohol solutions and critical-point dried with liquid carbon dioxide in a Leica Em CPD300 unit (Leica Mycrosystems, Vienna, Austria). The dried samples were mounted on specimen stubs with double-sided tape. The mounted tissues were gold-coated with a Quorum Q150R S sputter coated unit (Quorum Technologies, Ltd., Lewes, UK) and viewed with a variable-pressure microscope (Hitachi High Technologies Co., Ltd., Tokyo, Japan) at an accelerating voltage of 5 kV at the Institute of Marine Sciences of the Spanish Research Council (CSIC).

### 2.3. Setae Counting and Measurements

The number of setae was counted in each zone of the statocyst, and the results were averaged to obtain a mean number of setae for each of the five zones described.

To describe the diameter and length of the inner (ir) and outer (or) rows, these lengths were measured in 10 setae from the ir and or rows of the 40 statocysts (20 animals), and the results were averaged to obtain the mean size of the setae.

### 2.4. Transmission Electron Microscopy (TEM) Ultrastructural Analysis

For the TEM analysis, we used 5 animals (10 statocysts) of both sexes. The lobsters were anaesthetized and sacrificed with an overdose of 2-phenoxyethanol. The statocysts were dissected, fixed, and processed by routine procedures for analysis by TEM. Fixation was performed in 2.5% glutaraldehyde–2% paraformaldehyde for 24 h at 4 °C. Subsequently, the samples were osmicated in 1% osmium tetroxide, dehydrated in acetone, and embedded in Spurr. To orient the specimens properly, semithin sections (1 mm) were cut transversally or tangentially with a glass knife, stained with methylene blue, covered with Durcupan, and observed on an Olympus CX41 microscope. Ultrathin (around 100 nm) sections of the samples were then obtained by using a diamond knife (Diatome) with an Ultracut Ultramicrotome from Reichert-Jung. The sections were double-stained with uranyl acetate and lead citrate and viewed with a Jeol JEM 1010 microscope at 80 kV. Images were obtained with a Bioscan camera model 792 (Gatan, Pleasanton, CA, USA) at the University of Barcelona technical services.

## 3. Results

### 3.1. Scanning Electron Microscopy

**Figure 1:**
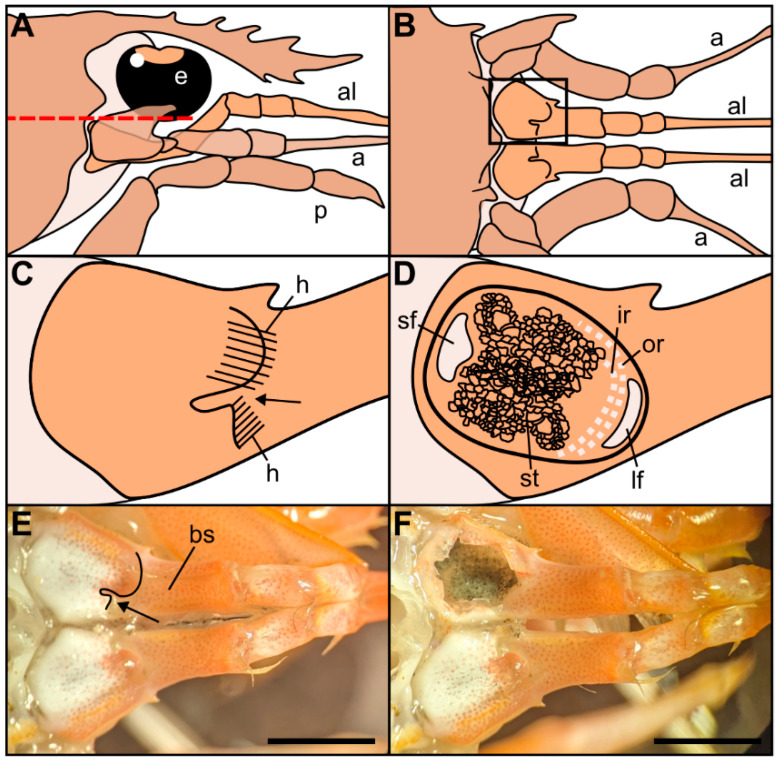
Drawings of the location and morphology of the statocyst organs in Norwegian lobster *Nephrops norvegicus*. (**A**) Right lateral view of the region of the statocysts, with the eye (e), antennule (al), antenna (a), pereiopod (p). (**B**) Dorsal view of the statocyst region after removal of the tissues that cover it dorsally, above the red dashed line in (**A**). (**C**) Detail from the inset in (**B**). Dorsal view of the closed statocyst in the basal segment of the antennule. Arrow: opening of the statocyst organ covered by hairs (h) on two sides. (**D**) Dorsal view of the opened statocyst in the basal segment of the antennule, showing the statolith (st) and the setae distribution: large setal field (lf), small setal field (sf), outer row setae (or), inner row setae (ir). (**E**) Dorsal view of the left and right antennules with the covered statocyst. Basal segment (bs). Arrow: statocyst’s opening. (**F**) Dorsal view of the left and right antennules with the left statocyst exposed. Note the statolith made of sand particles inside the statocyst. Scale bar: (**E**,**F**) = 5 mm.

**Figure 2:**
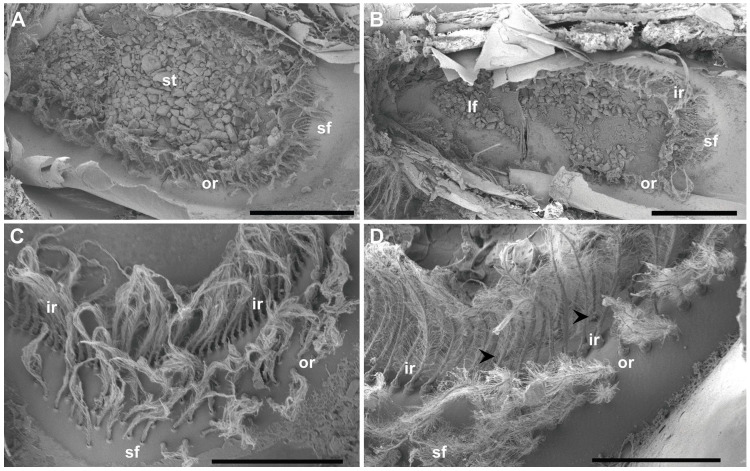
SEM. *N. norvegicus* statocyst structure and setae distribution. (**A**) Dorsal view of the statocyst with the statolith (st) covering all the sensilla. (**B**) Dorsal view of the statocyst after removing the statolith. The different areas are visible: inner single row (ir), outer double row (or), large setal field (lf), small setal field (sf). (**C**) Small setal field (sf) converging with the outer row (or). The inner row (ir) is visible behind it. (**D**) The arrowheads mark the short row that runs internally in parallel to the inner single row (ir) next to the small setal field (sf). Scale bars: (**A**,**B**) = 1 mm. (**C**) = 500 μm. (**D**) = 300 μm.

**Figure 3:**
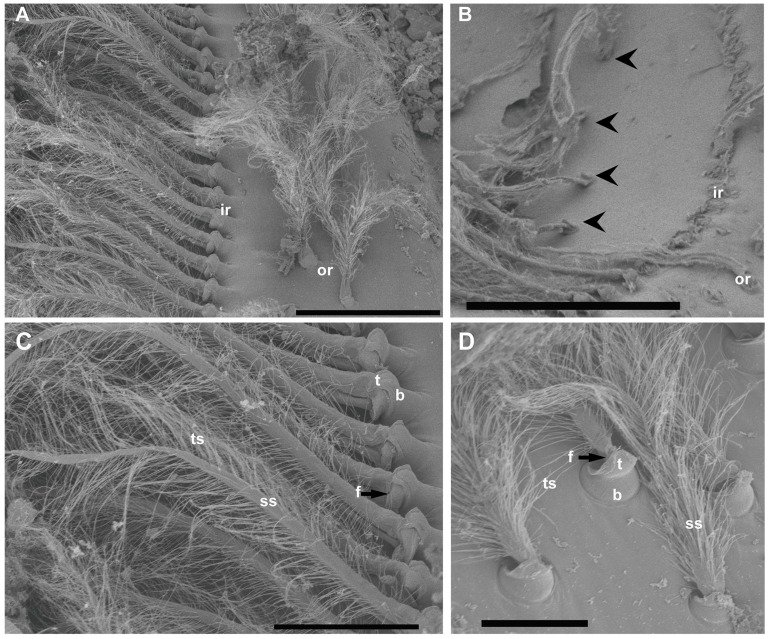
SEM. *N. norvegicus* statocyst setae structure. (**A**) Detail of the setae in the regular single inner row (ir) and the double outer row (or). (**B**) The arrowheads mark the short row visible after the removal of the inner row. (**C**) Inner row setae structure with a bulb (the proximal portion of the sensillum, b), a setal shaft (ss), a tooth (the smooth portion of the bulb, t), a fulcrum (a transverse fold, f), and filamentous thread-like strands (ts). (**D**) Setae structure in the small field. The setae in this area are similar to those described in (**C**) but with a shorter setal shaft (ss). Scale bars: (**A**) = 200 μm. (**B**) = 300 μm. (**C**) = 100 μm. (**D**) = 50 μm.

The statocyst, located on the basal segment of the antennule, appeared as a cup-like invagination of the cuticle forming a closed oval cavity (Figure 1). The ventral floor of the cavity had a depression that exhibited an inner cuticular sensory epithelium formed of hairs (mechanosensory setae) polarized towards the center and an overlying statoconia (consisting of sand granules cemented together by tegmental gland secretions (Figure 1D and Figure 2A), which stimulates the sensory hair cells (Figure 2 and Figure 3). After removing the sand granules (Figure 2B), the setae distribution within the statocyst was visible. A curved field consisting of an inner single row ($\bar{X}$ = 145, Figure 2B–D and Figure 3A–C) and an outer double row ($\bar{X}$ = 95, Figure 2A,D and Figure 3A) run on a circle around the medial and lateral rim of the central depression. On the posterior side, it merged into a small triangle of setae ($\bar{X}$ = 45, Figure 2C). A short row ($\bar{X}$ = 10, Figure 2D and Figure 3B) was shown to run internally in parallel to the inner single row only next to the small setal field. Opposite this small field, on the anterior side of the depression, there was a large setal triangular field ($\bar{X}$ = 125, Figure 2) ($\bar{X}$: setae mean number for N = 20). Table 1 reports the setae mean number for each statocyst region.

The inner row setae appeared overlaid by the statolith and hook-shaped, curving to the center (Figure 2 and Figure 3). The outer double row showed upright setae free of sand grains (Figure 2 and Figure 3). The structure of the setae in the two rows was similar, showing a bulb (the proximal portion of the sensillum) with a diameter of 30 μm at its widest point, a setal shaft extending for 370 μm (inner row) and 170 μm (outer row) into the statocyst lumen (setae mean measures for *N* = 20), a tooth (the smooth portion of the bulb), a fulcrum (a transverse fold), and filamentous hairs, which, in the inner row, showed attached statoconia (grain sands) (Figure 3C,D).

### 3.2. Transmission Electron Microscopy

**Figure 4:**
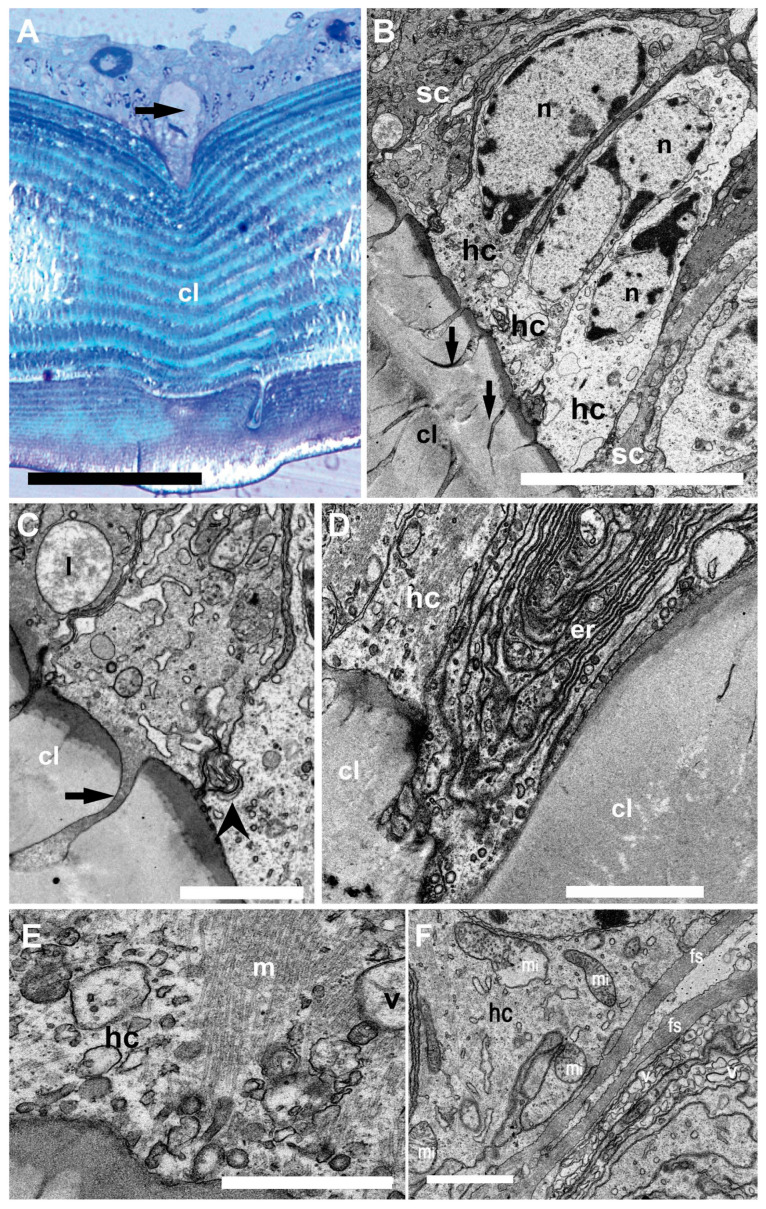
Light microscopy (**A**) and TEM (**B**–**F**). Inner structure of *N. Norvegicus* statocyst sensory epithelium. (**A**) The arrow marks the hair cell anchored in the cuticular layer (cl). (**B**) View of the sensory epithelium showing the hair cells (hc) buttressed by dark supporting cells (sc). Note the prominent nuclei (n) and the cytoplasm in the hair cells. The arrow marks the “anchor root” of the hair cell base in the cuticular layer (cl). (**C**) Detail of the anchoring structure of a hair cell (arrow). The arrowhead marks a complex membranous junction between two hair cells (lysosome (l)). (**D**) Abundant endoplasmic reticulum (er) is visible in a hair cell (hc) basal portion. (**E**) A bundle of microtubules (m) in relation with vesicles (v) in the basal portion of a hair cell. (**F**) Basal pole of a hair cell containing abundant mitochondria (mi). Note also fibrous strands (fs) and numerous vesicles (v). Scale bars: (**A**) = 100 µm. (**B**) = 10 µm. (**C**,**D**,**F**) = 2 µm. (**E**) = 1000 nm.

In the sensory epithelium (Figure 4), the statocyst hair cells appeared buttressed by dark supporting cells. The medial portion of the hair cells showed a clear cytoplasm containing large nuclei (Figure 4B). Towards the hair cell base, membranous junction complexes were shown to attach neighboring hair cells together. In addition, a root was found to anchor the cells into the cuticular layer.

The basal portion of the hair cells appeared to enter the cuticular layer and contain abundant endoplasmic reticulum and mitochondria. At this level, fibrous strands and numerous vesicles were shown.

## 4. Discussion

The statocyst is a crucial sensory system, the starting point of the sound perception process in marine invertebrates [12,13]. The outcome of this work is a description of the statocyst morphology in a previously undescribed Astacidea member. Astacidea is an infraorder of decapod crustaceans, including lobster and crayfish species. Although some previous works described the statocyst structure of some crayfish species, this is the first description of *N. norvegicus* statocyst, which allows a comparison to be made between some species.

The general morphology of the statocyst of *N. norvegicus* appeared to be similar to that of crayfish species previously described (*Procambarus clarkii* [24], *Faxonius limosus* [18], *Cherax destructor* [8]). Hertwig et al. [18] showed that all the setae of the statocyst capsule in *O. limosus* were morphologically identical. In *C. destructor* [8], the statocyst setae were of a single type too, and their morphology was closely similar to that of *O. limosus* setae [8]. However, despite these morphological similarities, the physiological responses of statocyst setae can differ [25]. Our results showed similarities in morphology between *N. norvegicus* setae and the previously described setae of the crayfish [8,18], even though these animals inhabit different ecosystems. In the next sections, we will attempt a further comparison on the arrangement of these elements in a variety of species with differing lifestyles, which has the potential to reveal principles of statocyst structure and function.

Hertwig et al. [18] found four distinct distribution areas of setae in *O. limosus* statocyst. Findley et al. [8] were able only to clearly detect three areas in *C. destructor* statocyst. Our results showed the presence of four areas, resembling the description of *O. limosus*. The inability to identify a fourth area in *C. destructor* might be due to the fact that the posterior line of eight setae (described in *O. limosus*) was not evident in *C. destructor*, but it is possible that these setae are included in the outer curved group. *N. norvegicus* exhibited similar numbers of setae as those in *C. destructor* and *O. limosus* in the different fields, confirming the similarity of the statocyst structure among *Astacidea* members, independently of their habitat.

In *N. norvegicus*, the curved and small fields appeared to occupy a similar position relative to the statolith, the same as that observed in *O. limosus* and *C. destructor*, and had approximately the same number of setae. As a consequence, it is probable that they serve similar functions about position information. Similarly, as in *C. destructor* and *O. limosus*, in lobsters (including our studied species, *N. norvegicus*) the lateral and posterior field respond to body roll, whereas the anterior field of setae responds mostly to acceleration [11].

It was hypothesized in different species that statolith-free setae are most suited for detecting angular accelerations [11,18,25]. In *N. norvegicus*, the double external row of setae is free from the statocyst; therefore, these setae could accomplish this function. Further research on behavioral aspects like movement in the water column or escape is necessary to determine the relation between these setae and the organization of the statocyst.

A description of the ultrastructure of the statocyst of the shrimp *Palaemon serratus* [6] confirms the specificity of the statocyst depending on its taxonomic group. *Palaemon* is a small species and, in addition to the obvious difference in the size of the setae with respect to *N. norvegicus*, its statocyst consists of vertical cellular projections arranged in a single row of hair cells oriented towards a common crescent-shaped central region and covered by a statolith (sand granules).

A comparison with taxonomically distant crustacean groups would lead us to see even greater differences, such as the simple crescent-shaped distribution of the few setae on the statocysts of the decapod Portunidae crabs (e.g., blue crab *Callinectes sapidus*) [14,19]. Interestingly, a recent description of the statocyst in the hermit crab *Dardanus calidus* (decapod, Anomura), taxonomically distant from *N. norvegicus*, showed four setal zones (medial, lateral, ventral, and caudal) as described in the present study, but compared to *N. norvegicus*, their distribution is different, and only the medial group of setae is in contact with the statolith [20].

The present TEM investigations essentially revealed that the statocyst hair cells have a robust microtubular cytoskeleton, are firmly anchored into the cuticular layer, and are strongly attached to one another. These morphological characteristics typically correspond to the organization of receptors devoted to the reception of mechanical vibrations. They actually attest to the ability of the organ to withstand water movements to some extent.

In decapod crustaceans, the setae have different functions depending on the species. In the crayfish *O. limosus* [18] and in the blue crab *C. sapidus* [14], the setae have a purely mechanical function. Shearing of the setae in one direction causes a mechanical constraint on a lever spring attached to the base of the setae, which in turn stimulates a sensory afferent neuron [12,26,27]. Similarly, in the crayfish *Astacus fluviatis*, setae stimulation causes mechanical stress in a chorda thread connected to a few bipolar afferent neurons [12,26,27]. In contrast, in the statocyst of the shrimp *P. serratus*, the setae have a mechanosensory function. The setae have a root buried in the cuticular layer that establishes synaptic contacts with infracuticular bipolar afferent neurons [6]. In the present study, a connection of the setae with neurons could not be observed. Based on the taxonomic proximity of the lobster to the crayfish and crab, the setae in *N. norvegicus* could have a mechanical function rather than a sensory function. Further ultrastructural research using TEM is needed to clarify the mode of connection between setae and afferent neurons in the statocyst of *N. norvegicus*.

## 5. Conclusions

*N. norvegicus* statocyst was described for first time. The statocyst setae appeared distributed in four areas (a curved field consisting of an inner single row and an outer double row that run on a circle around the medial and lateral rim of the central depression, a small setal field in the posterior part, a large setal field, opposite to the small field, and a short row running internally and lying parallel to the inner single row). The structure of the seta consists of a bulb (the proximal portion of the sensillum), a setal shaft, a tooth (the smooth portion of the bulb), and a fulcrum (a transverse fold), and filamentous hairs. The hair cells are firmly implanted within the cuticular layer. The data presented here contribute to the necessary knowledge of the sensory systems of crustaceans, as an initial step for the evaluation of the long-term effects of intense low-frequency sounds on their hearing ability. Although the type of innervation of the statocyst was not considered in the present study, the close taxonomic position of the lobster to that of the crayfish and crab would suggest that the setae in *N. norvegicus* are pure mechanoreceptors rather than sensory cells.

## Figures and Tables

**Table 1:** Summary of setae mean number in each statocyst region.

$\bar{X}$ (Setae Mean Number for *N* = 20)		Figure
Inner single row (ir)	145	Figure 2B–D and Figure 3A,C
Outer double row (or)	95	Figure 2A–D and Figure 3A
Large setal field (lf)	125	Figure 2B
Small setal field (sf)	45	Figure 2A–D
Short internal row	10	Figure 2D and Figure 3B

## Data Availability

Data are contained within the article.
